# Vitamin D concentrations and headache risk in children and adolescents aged 6 to 19 years: The mediating role of body mass index

**DOI:** 10.1097/MD.0000000000048477

**Published:** 2026-05-01

**Authors:** Min Zhang, Juan Xie, Cheng Guo, Tiesong Zhang, Kai Liu

**Affiliations:** a Department of Otolaryngology, Kunming Medical University Affiliated Children’s Hospital (Kunming Children’s Hospital), Kunming, Yunnan Province, China; b Yunnan Institute of Pediatrics, Yunnan Key Laboratory of Children’s Major Disease Research, Yunnan Province Clinical Research Center for Children’s Health and Disease, Kunming Medical University Affiliated Children’s Hospital (Kunming Children’s Hospital), Kunming, Yunnan Province, China; c Comprehensive Pediatrics, Kunming Medical University Affiliated Children’s Hospital (Kunming Children’s Hospital), Kunming , Yunnan Province, China.

**Keywords:** BMI, cross-sectional studies, headache, mediation analyses, vitamin D

## Abstract

Emerging evidence suggests vitamin D deficiency might be linked to increased headache risk, though consistent conclusions are lacking due to population and methodological heterogeneity. In addition, childhood and adolescent obesity may influence headache development through metabolic and inflammatory pathways, but the specific role of body mass index (BMI) in the relationship between vitamin D and headache is currently unclear. Therefore, this study used National Health and Nutrition Examination Survey (NHANES) large-scale population-based data to investigate the association between vitamin D levels and headache risk in children and adolescents aged 6 to 19 years and to analyze the possible mediating effect of BMI on this relationship. The aim of this study was to explore the complex association between vitamin D levels and headache. We analyzed 2 cycles of the NHANES dataset, which included a total of 7066 children and adolescents aged 6 to 19 years. Multivariate linear regression models, subgroup analyses and smoothed curve fitting were used to investigate the associations between vitamin D levels and headache, and the potential mediating role of BMI was explored. The results of the present study revealed a evident negative correlation between vitamin D levels and headache risk in children and adolescents, a finding that was further supported by smoothed curve fitting. Notably, this negative correlation was stronger in the female and adolescent groups. The results of the mediation analysis revealed that BMI had a evident mediating effect, with a mediation ratio of 20.94%. This study found that lower vitamin D levels were associated with a higher likelihood of headache in children and adolescents, and that BMI may play a partial mediating role. This finding provides new strategies for the prevention and treatment of headache in children and adolescents. More prospective studies are necessary to further validate this association and its underlying mechanisms.

## 1. Introduction

Headache is a common and significantly debilitating health issue among children and adolescents. Globally, up to 60% of children and adolescents suffer from severe headaches, with 7.7% to 9.1% of adolescents experiencing migraines.^[1,2]^ Frequent headaches or migraines not only impair daily functioning and academic performance but also markedly reduce quality of life and social interaction capabilities,^[3,4]^ making them a pressing public health concern. In recent years, the role of vitamin D in nonskeletal systems has garnered increasing attention, with its potential functions in neurology, immune regulation, and inflammation control being particularly noteworthy.^[5,6]^ Among neurological disorders, the association between vitamin D and headaches warrants special exploration. Some studies suggest that low vitamin D levels may be associated with an increased risk of headaches,^[7]^ and vitamin D supplementation may help alleviate headache symptoms.^[8,9]^ However, randomized controlled trials have also indicated that vitamin D supplementation does not significantly reduce headache incidence or severity.^[10]^ Consequently, the association between vitamin D and headaches in children and adolescents remains controversial, and its potential mechanisms are unclear.

Serum 25-hydroxyvitamin D, the primary biomarker for vitamin D status, is a fat-soluble vitamin obtained through sunlight exposure, dietary sources, and supplementation.^[11]^ Despite these multiple sources, vitamin D deficiency remains prevalent in the United States, particularly among children and adolescents.^[12-15]^ Addressing this deficiency in childhood may help prevent adverse health outcomes later in life. Vitamin D supplementation not only improves the frequency and intensity of headaches but also reduces the use of painkillers, preventive medications, and related side effect medications. Especially in the pediatric age group, where the use of medications is heavily burdened by ethical issues and side effects, the possibility of using milder interventions such as nutraceuticals like vitamin D could prove to be highly advantageous.^[16]^

Moreover, childhood obesity has emerged as a global health challenge,^[17,18]^ with vitamin D deficiency exhibiting a close association with obesity.^[19-21]^ Multiple cross-sectional and cohort studies indicate that childhood obesity correlates with an increased risk of headaches, while weight reduction contributes to the alleviation of headache symptoms.^[22-27]^ Against this backdrop, BMI may serve as a potential mediating factor in the relationship between vitamin D and headaches.

Based on current evidence, we hypothesized that lower vitamin D levels are associated with frequent or severe headaches in children and adolescents, with BMI potentially playing a role in this relationship. So, we used 2001 to 2004 nationally representative NHANES data and explored the relationship between vitamin D and headache in children and the potential mediating role of BMI via multivariate linear regression and mediation analyses. Due to the study’s cross-sectional design, causality couldn’t be confirmed. Future longitudinal and intervention studies are needed to validate these associations and explore potential mechanisms.

## 2. Materials and methods

### 2.1. Study population and data sources

**Figure 1.**
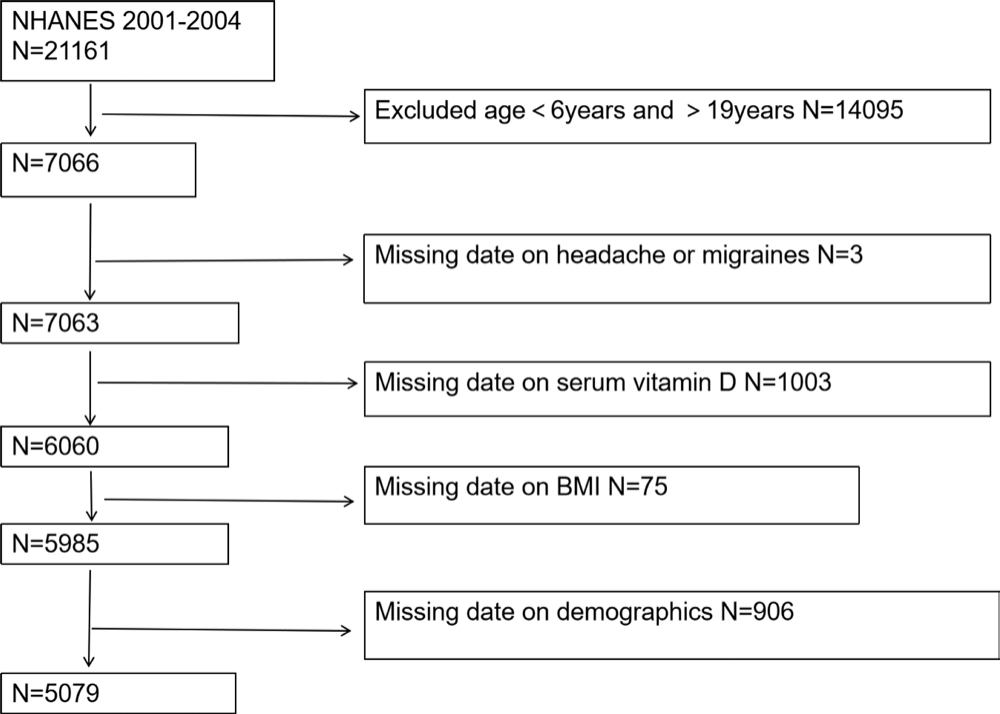
Flow chart of participant selection, NHANES 2001–2004. BMI = body mass index, NHANES = National Health and Nutrition Examination Survey.

The NHANES is a representative US national population survey that uses complex, multistage, and probability sampling methods to provide a wealth of information about the nutritional and health status of the general US population.^[28]^ The Ethics Review Board of the National Center for Health Statistics (NCHS) approved the study protocol. All survey participants signed a written informed consent form. Please visit https://wwwn.cdc.gov/nchs/nhanes/analyticguidelines.aspx for more information. This study utilized data from the NHANES 2001–2004 Continuous Survey and initially included a total of 21,161 participants. The study population consisted of 7066 children and adolescents aged 6 to 19 years. The following participants were excluded from the data analysis process: missing headache questionnaire information (n = 3), missing vitamin D testing data (n = 1003), missing BMI data (n = 75), or missing data on relevant covariates (n = 906). The final analysis included 5079 eligible participants, as shown in Figure 1.

Definition of headache: The primary outcome variable for this study was defined as the respondent’s affirmative answer to the following question on the NHANES questionnaire: “In the past 12 months, have you or the respondent experienced frequent or severe headaches, including migraines?”^[29]^

The serum vitamin D concentration was measured via the DiaSorin RIA kit and converted to an equivalent 25-hydroxyvitamin D concentration via the regression method via standardized liquid chromatography–tandem mass spectrometry.^[30]^ The same laboratory method (DiaSorin RIA) was used for vitamin D measurement across both survey cycles (2001–2004), and all values were standardized to LC-MS/MS equivalents to ensure consistency.

Centers for Disease Control and Prevention/National Centre for Health Statistics. Analytical notes for 25-hydroxyvitamin D data analyses via NHANES III (1988–1994), NHANES 2001–2006, and NHANES 2007–2010 (October 2015; https://wwwn.cdc.gov/nchs/nhanes/vitamind/analyticalnote.aspx?b=2013&e=2014&d=VID_H).

BMI is defined as BMI = weight (kg)/height (m)^2^.

### 2.2. Covariates

All covariates included in this study were selected a priori based on established associations with headache, vitamin D status, or BMI in the existing literature and clinical reasoning. No variables were added or removed based on the outcomes of the statistical models. Several potential confounding variables, including age, sex, race, education, poverty income ratio (PIR), total cholesterol (TC), high-density lipoprotein (HDL), C-reactive protein (CRP), bone alkaline phosphatase, hemoglobin, white blood cell count (WBC), neutrophil count, asthma, attention deficit, season of investigation, and presence of smokers in the household, were considered on the basis of published findings and clinical judgment. In logistic regression, age was considered a continuous variable, whereas in describing participant characteristics and subgroup analyses, age was categorized as 6 to 11 years and 12 to 19 years (defined as 6–11 years and 12 to 19 years for children and adolescents, respectively).^[31]^ In the descriptive participant characteristics and mediation analysis, BMI was treated as a continuous variable. The race categories included Mexican American, other Hispanic, non-Hispanic White, non-Hispanic Black, and other races (including multiracial). Asthma and attention deficit were defined as an affirmative response to the following question: “Have you ever been told you have asthma/attention deficit?”^[32,33]^ The presence of smokers in the household was defined as an affirmative answer to the question “Does anyone smoke in the home?” and a positive answer to this question. Since season affects vitamin D levels, we categorized testing time into 2 periods: November 1 to April 30 and May 1 to October 31.^[34]^

### 2.3. Statistical methods

This study was statistically analyzed via EmpowerStats software (version 4.1, http://www.empowerstats.com) and R software (version 3.4.3, http://www.R-project.org). Data for continuous variables are expressed as weighted means (95% CI), whereas categorical variables are expressed as percentages (95% CI). We compared the baseline characteristics of the headache population with those of the nonheadache population and assessed differences between the 2 groups via weighted *t* tests and chi-square tests. In our analysis, serum vitamin D was treated as a continuous variable for analysis. Participants were classified into quartiles (Q1–Q4) according to the sample distribution of vitamin D levels, without the application of clinical cutoff values. To investigate the association between vitamin D and headache, we developed 3 logistic regression models: model 1, not adjusted for any covariates; model 2, adjusted for age, sex, and race; and model 3, adjusted for age, sex, race, level of education, PIR, TC, HDL, bone alkaline phosphatase, CRP, WBC, neutrophil count, hemoglobin, asthma, attention deficit, survey season, and household smoking. In addition, we investigated the relationship between vitamin D and headache in different subgroups via subgroup analyses and interaction tests. Finally, a statistical mediation effect model with BMI as a mediating variable was developed via the bootstrapping method of the PROCESS macro (5000 replicate samples, 95% confidence intervals) to assess the indirect effect and mediation proportions of BMI in the relationship between vitamin D and headache risk.

### 2.4. Ethics guidelines

The NCHS Ethics Review Board reviewed and approved this study. Patients/participants provided written informed consent to participate in this study.

## 3. Results

### 3.1. Baseline data for selected populations

The study included 5079 participants, of whom 1210 (23.8%) reported headache. The headache group had a higher proportion of females. Compared with those of nonheadache patients, the age, BMI, neutrophil count and CRP values of headache patients were evidently greater (*P* < .05), whereas the vitamin D concentration, PIR value, hemoglobin and bone alkaline phosphatase level were evidently lower (*P* < .01), which may indicate potential nutritional and metabolic differences in the headache population (Table S1, Supplemental Digital Content, https://links.lww.com/MD/R773).

### 3.2. Analysis of the association between vitamin D and headaches

Table 1 shows the associations between vitamin D concentrations and headache in children and adolescents.

**Table 1:** Correlation studies between vitamin D and headache in children and adolescents.

	Model 1	Model 2	Model 3
	OR (95% CI) *P* value	OR (95% CI) *P* value	OR (95% CI) *P* value
Per 1 nmol/L vitamin D increase	0.99 (0.99, 0.99) <.0001	0.99 (0.99, 1.00) .0073	0.99 (0.99, 1.00) .0123
Categories (vitamin D)			
Q1	Ref.	Ref.	Ref.
Q2	0.77 (0.64, 0.93) .0060	0.92 (0.76, 1.13) .4262	0.94 (0.76, 1.14) .5158
Q3	0.65 (0.53, 0.79) <.0001	0.85 (0.68, 1.06) .1568	0.86 (0.68, 1.07) .1806
Q4	0.56 (0.46, 0.69) <.0001	0.74 (0.58, 0.93) .0097	0.75 (0.59, 0.95) .0185
*P* for trend	0.83 (0.77, 0.88) <.0001	0.91 (0.84, 0.98) .0095	0.91 (0.84, 0.98) .0169

Model 1: No covariates were adjusted.

Model 2: Age, sex, and race were adjusted.

Model 3: Age, sex, race, level of education, PIR, TC, HDL, bone alkaline phosphatase, CRP, WBC, neutrophil count, hemoglobin, asthma, attention deficit, survey season, and household smoking status were adjusted.

CI = confidence interval, CRP = C-reactive protein, HDL = high-density lipoprotein, OR = odds ratio, PIR = poverty income ratio, TC = total cholesterol, WBC = white blood cell count.

**Figure 2.**
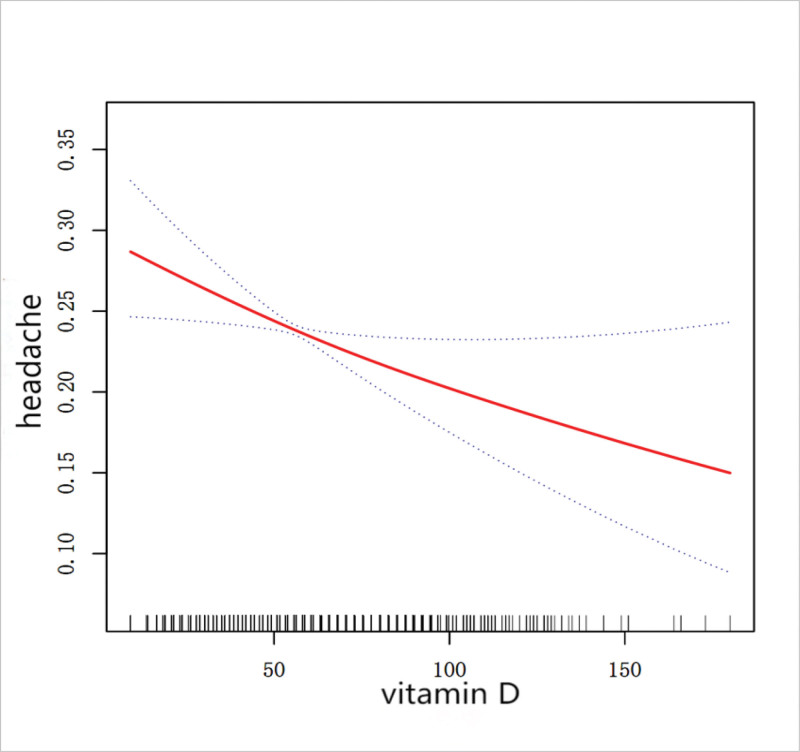
Vitamin D and headache smoothing curve fit.

We found that model 1 showed a 1% reduction in headache risk for each 1-unit increase in vitamin D; this negative correlation persisted in model 3,which fully adjusted for covariates, representing a weak yet statistically significant trend. After the vitamin D quartiles were grouped, participants in the highest quartile (Q4) demonstrated a evident inverse association with headache incidence in model 3 (Table 1). Linear regression analysis revealed a negative vitamin D-headache risk relationship, consistently supported by subsequent smoothed curve fitting (Fig. 2).

### 3.3. Subgroup analyses

Table 2 subgroup analyses showing the relationship between vitamin D and headache in different populations. Specifically, there were evident differences between vitamin D and headache among female, adolescent (12–19 years old), no asthma, no attention deficit, and Mexican American participants. Interaction tests revealed that the interaction effects of sex, age, asthma, attention deficit, and race on the relationship between vitamin D and headache were not evident (*P* > .05), suggesting that the negative association between vitamin D and headache was stable across subgroups of the population.

**Table 2:** Subgroup analysis.

Vitamin D		OR (95% CI) *P* value	*P* for interaction
Sex			.9657
Male	N = 2539	0.99 (0.99, 1.00) .0850	
Female	N = 2540	0.99 (0.99, 1.00) .0431	
Age (yr)			.6321
6–11	N = 1170	0.99 (0.98, 1.00) .1582	
12–19	N = 3909	1.00 (0.99, 1.00) .0274	
Asthma (%)			.5856
Yes	N = 842	0.99 (0.98, 1.00) .1103	
No	N = 4237	1.00 (0.99, 1.00) .0412	
Attention deficit (%)			.4256
			.5388
Yes	N = 4692	0.94 (0.84, 1.05) .2820	
No	N = 387	0.98 (0.96, 0.99) .0073	
Race (%)			.8308
Mexican American	N = 1566	0.99 (0.98, 1.00) .0931	
Other Hispanic	N = 194	0.99 (0.97, 1.01) .1550	
Non-Hispanic White	N = 1443	1.00 (0.99, 1.00) .3547	
Non-Hispanic Black	N = 1678	0.99 (0.99, 1.00) .1860	
Other races	N = 198	1.00 (0.97, 1.02) .6773	

CI = confidence interval, OR = odds ratio.

### 3.4. Mediation analysis of BMI

**Figure 3.**
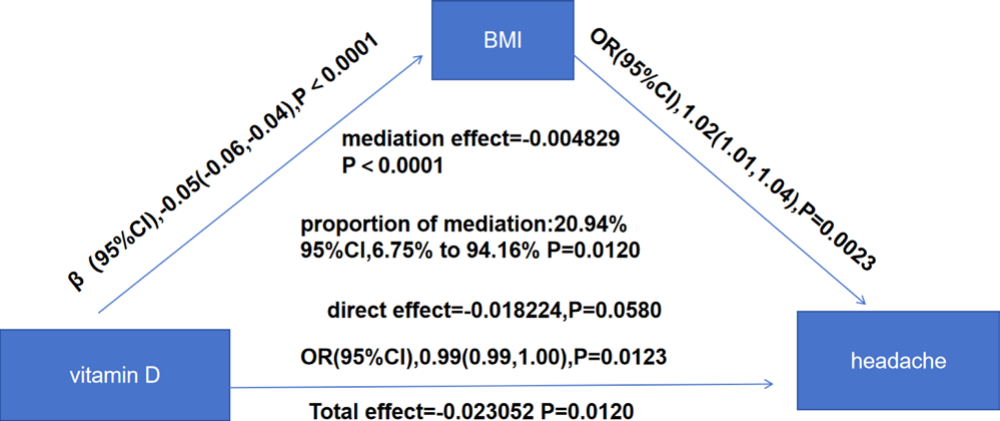
Mediation effect of BMI on the association between vitamin D and headache. Analysis of the effects of BMI on the associations between vitamin D and headache incidence. Sex, race, education level, PIR, total cholesterol (TC), high-density lipoprotein (HDL), bone alkaline phosphatase, C-reactive protein (CRP), white blood cell count (WBC), neutrophil count, hemoglobin, asthma, attention deficit, survey season, and household smoking status, were adjusted for. BMI = body mass index, CI = confidence interval, CRP = C-reactive protein, HDL = high-density lipoprotein, OR = odds ratio, PIR = poverty income ratio, TC = total cholesterol, WBC = white blood cell count.

We further investigated the potential mediating role of BMI in the correlation between vitamin D and headache risk. We found that higher vitamin D levels were associated with lower BMI (β = −0.05, 95% CI = −0.06, −0.04, *P* < .0001). Moreover, a higher BMI was associated with a greater risk of headache (OR = 1.02, 95% CI = 1.01, 1.04, *P* = .0023). The results suggest that BMI plays a partial mediating role. The *P* value for the mediating effect was .0120, and the mediation ratio was 20.94% (Fig. 3).

## 4. Discussion

Based on NHANES data, this study reveals an inverse association between vitamin D levels and headache risk in children and adolescents, particularly among females and adolescents. Although the effect size per unit increase in vitamin D was modest (OR = 0.99), it may hold public health relevance given the large sample. Mediation analysis further indicated that BMI partially explains this relationship, offering new insights into the role of vitamin D in pediatric headache.

Many clinical studies have suggested that vitamin D deficiency may increase the risk of headache in children.^[27,35-37]^ According to a retrospective analysis of Turkish children with headache, 69% had serum 25-hydroxyvitamin D levels < 50 nmol/L, suggesting that children with headache had low vitamin D levels.^[38]^ A cross-sectional study conducted in Norway confirmed that headache patients had lower mean vitamin D levels than did patients with other types of pain symptoms.^[39]^ In conclusion, a number of current observational and interventional studies support the existence of a correlation between vitamin D deficiency and headache in children. Our study also supports the conclusion that low levels of vitamin D increase the risk of headache. Unlike previous studies, we specifically examined children and adolescents across broad developmental stages, enabling more targeted treatment strategies that account for hormonal status and maturation.

To better understand the negative association between vitamin D and headache, the present study further analyzed the possible physiological mechanisms. First, reduced vitamin D levels correlate with elevated inflammatory markers such as CRP and tumor necrosis factor-α (TNF-α), which are directly implicated in headache pathogenesis through promoting neurogenic inflammation and sensitization of nociceptive pathways.^[40,41]^ In addition, studies have shown that vitamin D deficiency may be associated with neurotransmitter imbalance, increased neuroinflammation and neuronal damage in the brain, all of which are closely related to the development of headache.^[42]^ New research indicates that vitamin D also regulates the synthesis of 5-hydroxytryptamine, a key neurotransmitter that regulates pain and mood.^[43]^ In addition, vitamin D deficiency may lead to psychiatric disorders such as major and minor depression, schizophrenia, anxiety, and sleep disorders, further suggesting that vitamin D deficiency may promote or exacerbate headache through neurobiological and mental health pathways.^[44,45]^ Headaches are more common in children and adolescents with psychiatric disorders.^[46]^ Therefore, future studies should further focus on the overall role of vitamin D in inflammatory, neurological function, and mental health in order to develop more precise and comprehensive interventions.

Our subgroup analyses indicated a more robust association between vitamin D levels and headache risk among females. Several plausible biological mechanisms might underpin this sex-specific finding: First, estrogen may reduce bioavailable vitamin D by increasing vitamin D-binding protein concentrations, thereby limiting free circulating vitamin D^[47]^; second, females generally exhibit higher rates of vitamin D deficiency attributable to behaviors such as frequent sunscreen use, increased adiposity, and physiological states like pregnancy; third, females may possess greater neurological sensitivity to low vitamin D levels and exhibit enhanced pain perception pathways.^[48]^ Furthermore, the vitamin D-headache association was notably more pronounced in adolescents compared to younger children. Adolescents tend to engage more frequently in sedentary behaviors, such as prolonged screen time, which limit outdoor sunlight exposure essential for vitamin D synthesis.^[49]^ Additionally, rapid skeletal growth during adolescence heightens the physiological demand for vitamin D due to increased calcium utilization, potentially exacerbating relative vitamin D deficiency. Therefore, targeted monitoring and intervention strategies for vitamin D status should particularly prioritize adolescent and female populations.

In addition, this study clarified that BMI mediated 20.9% of the effect, partially explaining the relationship between vitamin D and headaches, thereby further elucidating the complex interplay between metabolic and nutritional factors. Obesity may reduce the bioavailability of vitamin D through adipose tissue hyperplasia while causing chronic inflammation and increasing headache risk.^[50]^ Furthermore, elevated BMI exacerbates chronic low-grade inflammation, heightening susceptibility to headaches. This finding is in line with the findings of another study that revealed a evidently increased risk of primary headache in a group of children with a BMI > 25,^[27]^ further suggesting a possible synergistic effect between obesity and vitamin D deficiency.

This study provides emerging evidence for the extraskeletal function of vitamin D but emphasizes the need to interpret the results in a specific context. Although many studies have focused on the potential clinical benefits of vitamin D supplementation for headache, our study revealed that interventions combining vitamin D supplementation with weight management may provide greater health benefits. Particularly in women and adolescents with headache, weight management and routine vitamin D screening may provide the basis for individualized treatment.

The present study has several limitations. First, owing to the cross-sectional design of the study, no clear causality could be derived. Second, using self-reported headache data from NHANES may involve recall bias, which could affect the accuracy of headache prevalence estimates. Furthermore, the NHANES lacked detailed data on headache subtypes and severity and did not consider the possible effects of other micronutrients. Finally, although the analyses were adjusted for multiple potential confounders, unknown or unmeasured confounders may still exist (such as sunlight exposure or diet). Therefore, future longitudinal studies and more in-depth nutrient analyses are necessary.

## 5. Conclusion

This study found that lower vitamin D levels were associated with higher headache prevalence, partly explained by BMI. However, more prospective studies are necessary to further validate this association and its underlying mechanisms.

## Author contributions

**Methodology:** Cheng Guo.

**Software:** Cheng Guo, Tiesong Zhang.

**Supervision:** Kai Liu.

**Writing – original draft:** Min Zhang, Juan Xie.

**Writing – review & editing:** Kai Liu.

## Supplementary Material

## References

[1] Abu-Arafeh I Razak S Sivaraman B Graham C. Prevalence of headache and migraine in children and adolescents: a systematic review of population-based studies. Dev Med Child Neurol. 2010;52:1088–97. 20875042 10.1111/j.1469-8749.2010.03793.x

[2] Wöber-Bingöl C. Epidemiology of migraine and headache in children and adolescents. Curr Pain Headache Rep. 2013;17:341. 23700075 10.1007/s11916-013-0341-z

[3] Schiller K Schiller V Kortas A. Primary headache is related to reduced health-related quality of life in children with epilepsy. Healthcare (Basel). 2024;12:426. 38391802 10.3390/healthcare12040426 PMC10887633

[4] Sharma A Khurana P Venkatraman A Gupta M. Subsume pediatric headaches in psychiatric disorders? Critiques on delphic nosology, diagnostic conundrums, and variability in the interventions. Curr Pain Headache Rep. 2024;28:651–62. 38367199 10.1007/s11916-024-01225-7

[5] Anjum I Jaffery SS Fayyaz M Samoo Z Anjum S. The role of vitamin D in brain health: a mini literature review. Cureus. 2018;10:e2960. 30214848 10.7759/cureus.2960 PMC6132681

[6] Jeon SM Shin EA. Exploring vitamin D metabolism and function in cancer. Exp Mol Med. 2018;50:1–14. 10.1038/s12276-018-0038-9 PMC5938036 29657326

[7] Prakash S Mehta NC Dabhi AS Lakhani O Khilari M Shah ND. The prevalence of headache may be related with the latitude: a possible role of Vitamin D insufficiency? J Headache Pain. 2010;11:301–7. 20464624 10.1007/s10194-010-0223-2 PMC3476351

[8] Al-Nimer MS. Vitamin D: Is it a primary hormone targeting the migraine headache or just as adjunct therapy? Neurosciences (Riyadh). 2017;22:69. 28064336 10.17712/nsj.2017.1.20160561 PMC5726843

[9] Yang Y Zhang HL Wu J. Is headache related with vitamin D insufficiency? J Headache Pain. 2010;11:369; author reply 371. 20602247 10.1007/s10194-010-0235-y PMC3476355

[10] Knutsen KV Madar AA Brekke M. Effect of vitamin D on musculoskeletal pain and headache: A randomized, double-blind, placebo-controlled trial among adult ethnic minorities in Norway. Pain. 2014;155:2591–8. 25261164 10.1016/j.pain.2014.09.024

[11] Sailike B Onzhanova Z Akbay B Tokay T Molnár F. Vitamin D in central nervous system: implications for neurological disorders. Int J Mol Sci . 2024;25:7809. 39063051 10.3390/ijms25147809 PMC11277055

[12] Kumar J Muntner P Kaskel FJ Hailpern SM Melamed ML. Prevalence and associations of 25-hydroxyvitamin D deficiency in US children: NHANES 2001-2004. Pediatrics. 2009;124:e362–70. 19661054 10.1542/peds.2009-0051 PMC3749840

[13] Mansbach JM Ginde AA Camargo CA. Serum 25-hydroxyvitamin D levels among US children aged 1 to 11 years: do children need more vitamin D? Pediatrics. 2009;124:1404–10. 19951983 10.1542/peds.2008-2041 PMC3765249

[14] Saintonge S Bang H Gerber LM. Implications of a new definition of vitamin D deficiency in a multiracial us adolescent population: the National Health and Nutrition Examination Survey III. Pediatrics. 2009;123:797–803. 19255005 10.1542/peds.2008-1195

[15] Gordon CM DePeter KC Feldman HA Grace E Emans SJ. Prevalence of vitamin D deficiency among healthy adolescents. Arch Pediatr Adolesc Med. 2004;158:531–7. 15184215 10.1001/archpedi.158.6.531

[16] Dell’Isola GB Tulli E Sica R. The vitamin D role in preventing primary headache in adult and pediatric population. J Clin Med. 2021;10:5983. 34945279 10.3390/jcm10245983 PMC8709239

[17] DiPietro L Mossberg HO Stunkard AJ. A 40-year history of overweight children in Stockholm: life-time overweight, morbidity, and mortality. Int J Obes Relat Metab Disord. 1994;18:585–90. 7812410

[18] Nieto FJ Szklo M Comstock GW. Childhood weight and growth rate as predictors of adult mortality. Am J Epidemiol. 1992;136:201–13. 1415142 10.1093/oxfordjournals.aje.a116486

[19] Marquina C Mousa A Scragg R de Courten B. Vitamin D and cardiometabolic disorders: a review of current evidence, genetic determinants and pathomechanisms. Obes Rev. 2019;20:262–77. 30450683 10.1111/obr.12793

[20] Cheng S Massaro JM Fox CS. Adiposity, cardiometabolic risk, and vitamin D status: the Framingham heart study. Diabetes. 2010;59:242–8. 19833894 10.2337/db09-1011 PMC2797928

[21] Hyppönen E Boucher BJ. Adiposity, vitamin D requirements, and clinical implications for obesity-related metabolic abnormalities. Nutr Rev. 2018;76:678–92. 30020507 10.1093/nutrit/nuy034

[22] Pinhas-Hamiel O Frumin K Gabis L. Headaches in overweight children and adolescents referred to a tertiary-care center in Israel. Obesity (Silver Spring). 2008;16:659–63. 18239560 10.1038/oby.2007.88

[23] Hershey AD Powers SW Nelson TD; American Headache Society Pediatric Adolescent Section. Obesity in the pediatric headache population: a multicenter study. Headache. 2009;49:170–7. 18783447 10.1111/j.1526-4610.2008.01232.x

[24] Robberstad L Dyb G Hagen K Stovner LJ Holmen TL Zwart JA. An unfavorable lifestyle and recurrent headaches among adolescents: the HUNT study. Neurology. 2010;75:712–7. 20720191 10.1212/WNL.0b013e3181eee244

[25] Ravid S Shahar E Schiff A Gordon S. Obesity in children with headaches: association with headache type, frequency, and disability. Headache. 2013;53:954–61. 23574609 10.1111/head.12088

[26] Lu SR Fuh JL Wang SJ. Incidence and risk factors of chronic daily headache in young adolescents: a school cohort study. Pediatrics. 2013;132:e9–e16. 23776112 10.1542/peds.2012-1909

[27] Hanci F Kabakuş N Türay S Bala KA Dilek M. The role of obesity and vitamin D deficiency in primary headaches in childhood. Acta Neurol Belg. 2020;120:1123–31. 30963478 10.1007/s13760-019-01134-2

[28] Curtin LR Mohadjer LK Dohrmann SM. The national health and nutrition examination survey: sample design, 1999-2006. Vital Health Stat 2. 2012:1–39. 22788053

[29] Zhu B Zhao R Wang L. Gender-specific inflammatory burden and headache risk in youth: a NHANES analysis. Head Face Med. 2024;20:71. 39633488 10.1186/s13005-024-00475-5 PMC11619679

[30] Zhang X Wu J Wu T Guo L Zhang R Jin X. Correlation between 25-hydroxyvitamin D and severe headache or migraine: evidence from NHANES database. Food Nutr Res. 2024;68:10338. 10.29219/fnr.v68.10338 PMC11650721 39691689

[31] Xiao L Yang C Gu W Liu R Chen D. Associations between serum copper, zinc, selenium level and sex hormones among 6-19 years old children and adolescents in NHANES 2013-2016. Front Endocrinol (Lausanne). 2022;13:924338. 36171898 10.3389/fendo.2022.924338 PMC9511025

[32] Sun T Fan K Han Z Qiao H. Dose-response relationship between the fatty liver index and asthma risk: NHANES 2001~2018. Endocr J. 2025;72:229–37. 39537178 10.1507/endocrj.EJ24-0248 PMC11850101

[33] Chopra V Harley K Lahiff M Eskenazi B. Association between phthalates and attention deficit disorder and learning disability in U.S. children, 6-15 years. Environ Res. 2014;128:64–9. 24267794 10.1016/j.envres.2013.10.004 PMC3889659

[34] Michaëlsson K Wolk A Byberg L Mitchell A Mallmin H Melhus H. The seasonal importance of serum 25-hydroxyvitamin D for bone mineral density in older women. J Intern Med. 2017;281:167–78. 27665750 10.1111/joim.12563

[35] Kiliç B Kiliç M. Evaluation of vitamin D levels and response to therapy of childhood migraine. Medicina (Kaunas). 2019;55:321. 31261815 10.3390/medicina55070321 PMC6681503

[36] Tozzi E Boncristiano A Antenucci A, Di Loreto S Farello G. P013. 25(OH)D Level and headache in children sample. J Headache Pain. 2015;16(Suppl 1):A84. 28132304 10.1186/1129-2377-16-S1-A84 PMC4759109

[37] Gallelli L Michniewicz A Cione E. 25-hydroxy vitamin D detection using different analytic methods in patients with migraine. J Clin Med. 2019;8:895. 31234518 10.3390/jcm8060895 PMC6617382

[38] Donmez A Orun E Sonmez FM. Vitamin D status in children with headache: a case-control study. Clin Nutr ESPEN. 2018;23:222–7. 29460803 10.1016/j.clnesp.2017.09.010

[39] Knutsen KV Brekke M Gjelstad S Lagerløv P. Vitamin D status in patients with musculoskeletal pain, fatigue and headache: a cross-sectional descriptive study in a multi-ethnic general practice in Norway. Scand J Prim Health Care. 2010;28:166–71. 20642395 10.3109/02813432.2010.505407 PMC3442332

[40] Filgueiras MS Rocha NP Novaes JF Bressan J. Vitamin D status, oxidative stress, and inflammation in children and adolescents: a systematic review. Crit Rev Food Sci Nutr. 2020;60:660–9. 30596263 10.1080/10408398.2018.1546671

[41] Ghorbani Z Togha M Rafiee P. Vitamin D3 might improve headache characteristics and protect against inflammation in migraine: a randomized clinical trial. Neurol Sci. 2020;41:1183–92. 31897949 10.1007/s10072-019-04220-8

[42] Menéndez SG Manucha W. Vitamin D as a modulator of neuroinflammation: implications for brain health. Curr Pharm Des. 2024;30:323–32. 38303529 10.2174/0113816128281314231219113942

[43] Hao S Shi W Liu W Chen QY Zhuo M. Multiple modulatory roles of serotonin in chronic pain and injury-related anxiety. Front Synaptic Neurosci. 2023;15:1122381. 37143481 10.3389/fnsyn.2023.1122381 PMC10151796

[44] Lally J Gaughran F. Vitamin D in schizophrenia and depression: a clinical review. BJPsych advances. 2019;25:240–8.

[45] Garcion E Wion-Barbot N Montero-Menei CN Berger F Wion D. New clues about vitamin D functions in the nervous system. Trends Endocrinol Metab. 2002;13:100–5. 11893522 10.1016/s1043-2760(01)00547-1

[46] Polese D Belli A Esposito D. Psychological disorders, adverse childhood experiences and parental psychiatric disorders in children affected by headache: a systematic review. Neurosci Biobehav Rev. 2022;140:104798. 35907492 10.1016/j.neubiorev.2022.104798

[47] Powe CE Evans MK Wenger J. Vitamin D-binding protein and vitamin D status of black Americans and white Americans. N Engl J Med. 2013;369:1991–2000. 24256378 10.1056/NEJMoa1306357 PMC4030388

[48] Manson JE. Pain: sex differences and implications for treatment. Metabolism. 2010;59:S16–20. 20837187 10.1016/j.metabol.2010.07.013

[49] Matthews CE Chen KY Freedson PS. Amount of time spent in sedentary behaviors in the United States, 2003-2004. Am J Epidemiol. 2008;167:875–81. 18303006 10.1093/aje/kwm390 PMC3527832

[50] Szymczak-Pajor I Miazek K Selmi A Balcerczyk A Śliwińska A. The action of vitamin d in adipose tissue: is there the link between vitamin D deficiency and adipose tissue-related metabolic disorders? Int J Mol Sci . 2022;23:956. 35055140 10.3390/ijms23020956 PMC8779075

