# Supplementary material for: Vitamin D concentrations and headache risk in children and adolescents aged 6 to 19 years: The mediating role of body mass index
Source: Medicine (Baltimore). 2026 May 1;105(18):e48477. doi: 10.1097/MD.0000000000048477 (PMC13138427; doi:10.1097/MD.0000000000048477)
Supplement: Supplementary file 1 [file medi-105-e48477-s001.pdf]

Vitamin D concentrations and headache risk in children and adolescents aged 6–19 years: the mediating role of body mass index

Min Zhang M.S.1#, Juan Xie M.S.2#, Cheng Guo M.S.2, Tiesong Zhang Ph.D.1, Kai Liu Ph.D.2*

Author Affiliations:

1. Department of Otolaryngology, Kunming Children's Hospital, Kunming, Yunnan Province, China.

2. Comprehensive pediatrics, Kunming Children's Hospital, Kunming, Yunnan Province, China.

#These authors contributed equally to the work and should be considered co-first authors.

*Corresponding Author:

Kai Liu Ph.D. Comprehensive Pediatrics & Pulmonary and Critical Care Medicine, Kunming Children’s Hospital, Shulin Street 28, Kunming, China, 650103.

Email: liukai@kmmu.edu.cn

Supplementary table 1 Baseline characteristics of the NHANES 2001-2004 participants

Characteristics a	Non-headache (3869)	Headache (1210)	Standardize diff.	P-value
N	3869	1210		
Age(year)	13.88 ± 3.29	14.73 ± 2.98	0.27 (0.21, 0.34)	<0.001
Vitamin D Concentration（nmol/l）			0.21 (0.14, 0.27)	<0.001
Q1	840 (21.71%)	357 (29.50%)		
Q2	959 (24.79%)	314 (25.95%)		
Q3	1007 (26.03%)	283 (23.39%)		
Q4	1063 (27.47%)	256 (21.16%)		
PIR	2.09 ± 1.54	1.94 ± 1.51	0.09 (0.03, 0.16)	0.005
WBC （1000 cells/uL）	6.91 ± 2.09	7.02 ± 2.17	0.05(-0.01,0.12)	0.102
Neutrophils（1000 cells/uL）	3.83 ± 1.75	3.96 ± 1.79	0.07 (0.00, 0.13)	0.034
Hemoglobin(g/dl)	13.98 ± 1.35	13.83 ± 1.39	0.11 (0.04, 0.17)	<0.001
BMI(kg/m2)	22.45 ± 5.68	23.99 ± 6.27	0.26 (0.19, 0.32)	<0.001
CRP(mg/dl)	0.17 ± 0.47	0.22 ± 0.49	0.09 (0.02, 0.15)	0.007
Bone.alkaline. phosphotase (ug/l)	62.27 ± 43.67	51.85 ± 41.66	0.24 (0.18, 0.31)	<0.001
TC(mmol/l)	163.83 ± 31.17	165.34 ± 31.08	0.05(-0.02,0.11)	0.141
HDL(mmol/l)	52.29 ± 12.28	51.69 ± 12.90	0.05(-0.02,0.11)	0.143
Sex			0.19 (0.12, 0.25)	<0.001

Male	2019 (52.18%)	520 (42.98%)		
Female	1850 (47.82%)	690 (57.02%)		
Race			0.16 (0.10, 0.23)	<0.001
MexicanAmerican	1226 (31.69%)	340 (28.10%)		
Other Hispanic	137 (3.54%)	57 (4.71%)		
Non-Hispanic White	1139 (29.44%)	304 (25.12%)		
Non-Hispanic Black	1219 (31.51%)	459 (37.93%)		
Other Races	148 (3.83%)	50 (4.13%)		
Education level			0.15 (0.09, 0.22)	<0.001
Below high school	2707 (69.97%)	763 (63.06%)		
High school graduation or equivalent	1004 (25.95%)	396 (32.73%)		
Above high school	158 (4.08%)	51 (4.21%)		
Survey season			0.02(-0.04,0.09)	0.501
1 November to 30 April	2026 (52.36%)	647 (53.47%)		
1 May to 31 October	1843 (47.64%)	563 (46.53%)		
Household Smokers			0.12 (0.06, 0.19)	<0.001
Yes	825 (21.32%)	321 (26.53%)		

No	3044 (78.68%)	889 (73.47%)		
asthma			0.15 (0.09, 0.22)	<0.001
Yes	588 (15.20%)	254 (20.99%)		
NO	3281 (84.80%)	956 (79.01%)		
Attention deficit			0.14 (0.07, 0.20)	<0.001
Yes	259 (6.69%)	128 (10.58%)		
No	3610 (93.31%)	1082 (89.42%)		

All values are presented as mean ± SD or as counts (weighted, proportion). PIR,poverty income ratio;WBC,white blood count;BMI,body mass index;CRP,C-Reactive Protein;TC,Total Cholesterol;HDL,high-density lipoprotein.
